# Women’s empowerment and elective cesarean section for a single pregnancy: a population-based and multivariate study in Vietnam

**DOI:** 10.1186/s12884-020-03482-x

**Published:** 2021-01-04

**Authors:** Myriam de Loenzien, Quoc Nhu Hung Mac, Alexandre Dumont

**Affiliations:** 1 Research Institute for Sustainable Development (IRD)-Centre Population and Development (CEPED), INSERM ERL 1244, Université de Paris, 45 rue des saints pères, 75006 Paris, France; 2 Pham Ngoc Thach University of Medicine, 2 Duong Quang Trung, P. 12, Q. 10, Ho Chi Minh City, Vietnam

**Keywords:** Childbirth, women’s empowerment, cesarean section, Vietnam, logistic regression analysis, parity, biomedicalization, population

## Abstract

**Background:**

Women’s empowerment, and maternal and neonatal health are important targets of the Sustainable Development Goals. Our objective is to examine the relationship between women’s empowerment and elective cesarean section (ECS), focusing on Vietnam, a country where the use of CS has increased rapidly in recent decades, which raises public health concerns.

**Methods:**

We hypothesized that in the context of the developing biomedicalization of childbirth, women’s empowerment increases the use of ECS due to a woman’s enhanced ability to decide her mode of delivery. By using microdata from the 2013–2014 Multiple Indicator Clusters Survey, we conducted a multivariate analysis of the correlates of ECS. We studied a representative sample of 1343 institutional single birth deliveries. Due to higher ECS rates among multiparous (18.4%) than primiparous women (10.1%) and the potential interaction between parity and other correlates, we used separate models for primiparous and multiparous women.

**Results:**

Among the indicators of women’s external resources, which include a higher level of education, having worked during the previous 12 months, and having one’s own mobile phone, only education differed between primiparous and multiparous women, with a higher level among primiparous women. Among primiparous women, no resource indicator was significantly linked to ECS. However, considering women’s empowerment facilitated the identification of the negative impact of having had fewer than 3 antenatal care visits on the use of ECS.

Among multiparous women, disapproval of intimate partner violence (IPV) was associated with a doubled likelihood of undergoing ECS (odds ratio = 2.415), and living in an urban area also doubled the likelihood of ECS. The positive association with living in the richest household quintile was no longer significant when attitude towards IPV was included in the model. In both groups, being aged 35 or older increased the likelihood of undergoing ECS, and this impact was stronger in primiparous women.

**Conclusions:**

These results underline the multidimensionality of empowerment, its links to other correlates and its contribution to clarifying the influence of these correlates, particularly for distinguishing between medical and sociocultural determinants. The results advocate for the integration of women's empowerment into policies aimed at reducing ECS rates.

## Background

Women’s empowerment and perinatal health are two important targets of the 2030 Sustainable Development Goals [1]. Cesarean section (CS) is necessary when a vaginal delivery increases the risk of poor outcomes for the newborn and/or the mother and has no medical benefit when it is not medically indicated [2]. Furthermore, the overuse of CS may add an economic burden, especially in low- and middle-income countries [3]. The overall rate of CS has reached high levels in many countries [4, 5], especially in middle-income countries [6]. This trend signifies an increasing practice of non-medically indicated CS deliveries. This increase raises concerns, as the average proportion of women who need a CS has been estimated to be approximately 10–15% of the population [7]. Furthermore, previous studies have suggested that a rate of over 20% does not provide better outcomes for maternal and neonatal health [8].

CS can be decided before the onset of labor (elective CS (ECS)) or during labor (emergency CS). ECS results from a decision process that involves the healthcare worker and, to some extent, the pregnant women and her relatives, while women have less opportunity to participate in the decision to deliver by CS during labor due to the urgency of the procedure. ECS and emergency CS may be used for the safety of the mother and the newborn. Although obstetricians tend to prioritize medical indications and, when possible, follow women’s preferences, they may also give priority to ECS for nonmedical reasons such as organizational, administrative, technical, financial or social advantages [9].

Women’s empowerment has been defined as a multilevel and dynamic process of transitioning from the *existence* of reproductive choices partly shaped by women’s motivations, to women’s *exercise* of choice by which women use negotiation, access to information, communication, decision making, and self-efficacy to overcome barriers and obstacles such as economic costs, fears, pressures from healthcare workers and family members, and, finally, to the *achievement* of choice [10]. Empowerment can be modeled as a three-dimensional process that includes enabling resources, instrumental agency and intrinsic agency [11]. Agency expresses a woman’s ability to make choices that pertain to her life, act in line with her choices and behave consistently with achieved choices [10]. Instrumental agency is often measured as a woman’s ability to make household and family-level decisions, whereas intrinsic agency is measured as the extent to which her expression of gender attitudes reflects or rejects normative beliefs [11].

Little information is available on the influence of women’s empowerment on the mode of childbirth [12]. In most studies, the social context appears to be a crucial determinant of the impact of empowerment on women’s health and well-being throughout the childbirth process [13–15]. One study that focused on vaginal delivery demonstrated the difficulty of ensuring that women’s autonomy is respected and the crucial role of women’s empowerment in childbirth regarding access to information, the presentation of self-assertion and a woman’s relationship with midwives [16]. One cause of high rates of CS that is linked to women’s empowerment is women’s request for CS, which reaches a high level in middle-income countries [17]. Several reasons have been mentioned for this. One of these reasons lies in the biomedicalization of childbirth, which has been increasing rapidly in recent decades [18]. In this context, the higher price of CS compared to that of vaginal delivery may contribute to enhancing the social value attached to CS because it is associated with being more privileged.

The objective of this study is to examine the relationship between women’s empowerment and the use of ECS in Vietnam by using the Vietnam national survey database. In this country, the proportion of CS among all deliveries has increased rapidly, from 20.0% in 2011 to 27.5% in 2014 [19], which raises concern [20]. ECS represented more than half of all CS and 14.2% of all births in 2014 [21]. A high level of childbirth biomedicalization contributes to this trend in the context of persistent gender inequality despite political commitment to women’s empowerment [22, 23]. Qualitative studies emphasize the fear of childbirth as a major determinant of maternal requests for CS in the absence of effective pharmacological pain relief and a lack of social support during labor [24, 25]. Empowered women may be more able to request CS. Parity may play a specific role, as women who have previously delivered vaginally can rely on their experience. However, women who have had CS tend to be assigned to this mode of delivery despite the potential multiple risks involved [19]. Our hypothesis is that in the context of biomedicalization marked by the high value of CS assigned to doctors and modern medicine and a patriarchal society that involves many constraints for women in their social and economic activities, women’s empowerment, including their attitudes in favor of gender equality and their external resources and opportunity structures, fosters a recourse to ECS, with contrasting impacts according to parity.

## Methods

### Data

To allow for comparisons, we used a methodology similar to that of a previous study performed by the authors on CS in Vietnam, using “data from the Multiple Indicator Clusters Survey (MICS) conducted from December 2013 to April 2014 by the General Statistical Office in collaboration with the United Nations Children’s Fund (UNICEF)” [26]. We included women who delivered in an institutional setting and excluded multiple pregnancies because they often have a high risk of undergoing medically indicated CS due to abnormal presentation or fetal growth restriction. We focused on women whose last childbirth occurred during the 2 years prior to the survey. Overall, 1343 women were included in this study. Among them, 578 were primiparous, and 765 were multiparous.

### Outcome measures and covariates

The outcome variable under study was the mode of delivery. This variable took 1 if a woman had decided on ECS before the onset of labor and 0 if she had a vaginal delivery after a trial of labor or if she had an emergency CS.

Among the covariates, we paid specific attention to the following four indicators (Table 1): the woman’s highest level of education (primary or less, secondary, and tertiary including college and university); her activity for wages or a salary during the previous 12 months (she worked versus she had not worked); her ownership of a mobile phone (she had versus she did not have her own mobile phone); and her attitude towards intimate partner violence (IPV; she declared that a husband is justified in beating or hitting his wife in at least one of the circumstances cited versus she declared that in none of these circumstances is the husband justified in beating or hitting his wife). To explore situations related to the topics linked to having to decide on a mode of delivery, the two circumstances referred to in this question are (1) the wife goes out without telling her husband, and (2) the wife argues with her husband.

The covariates included the qualifications of the antenatal healthcare provider (doctor or other type of provider) and the number of antenatal care (ANC) visits. Given that the 2016 Vietnamese guidelines recommend at least four ANC visits [27] and that the standard ANC model recommends at least eight ANC visits [28], we compare women who had 3 or fewer visits, women who had 4 to 7 visits and women who had 8 or more visits. Given that the age of 35 years is explicitly used as a risk factor for medically indicated ECS [27], we distinguished among women who delivered as teenagers, those who delivered between 20 and 34 years and those who delivered at age 35 and over. Other covariates included the sector of the place of delivery (public versus private healthcare sector), the size of the newborn as declared by the mother, the sex of the newborn, the quintile of the wealth of the household (poorest, poor, middle, rich or richest), the ethnicity of the household head (Kinh and Hoa ethnic groups compared with minority ethnic groups), and the region (North-Central, Mekong River Delta, Red River Delta, Northern Midlands, Central Highlands or the Southeast).

### Statistical analysis

Given the potential discriminant effect of parity, we separately analyzed primiparous and multiparous women. For each group, we conducted a bivariate analysis that used relative frequencies and performed a stepwise logistic regression to assess the characteristics associated with ECS as opposed to a trial of labor. As was the case in the previous study, “the bivariate analyses used the women’s sample weights” [26]. For the comparison between groups, a chi-square test was used. We used a multivariate logistic regression with a complex sampling general linear fixed effects model that included a replacement sampling procedure. Two strata were considered: the cluster and the household. The significance level was adjusted by using the least significant difference. We calculated odds ratios (ORs) with a 95% confidence interval. We used a finite population correction when estimating variance under a simple random sampling assumption.

The logistic regression models included only the variables that proved to have a significant effect in the bivariate analysis at the lower level of risk (*p* < 0.05). The higher level of risk (*p* < 0.10) was used as additional information for bivariate analyses. The first multivariate model did not include the empowerment variables, whereas the second model included the same characteristics as the first model with the addition of the indicators that were significantly linked to women’s rates of ECS in at least one of the parity groups in the bivariate analysis. We performed all statistical analyses with IBM PASW Statistics 18 software at Paris Descartes University.

### Ethics and consent to participate

As stated in previous research works on the database used, “the Vietnam General Statistics Office (GSO) and UNICEF approved the tools of the Vietnam MICS before the survey was conducted according to the ethical standards in the 1964 Declaration of Helsinki and its later amendments or comparable ethical standards. The MICS data are freely available through the UNICEF MICS website, and there is no need to obtain ethical approval before using the data” [26].

## Results

The level of empowerment was high for all indicators (Table 1). More than one-quarter of the women had a tertiary level of education (25.3%), and more than half of them had a secondary level of education (61.8%). Most of the women had worked for wages or a salary in the previous 12 months (81.2%). An even higher proportion of the women had their own mobile phone (88.7%). In addition, the women overwhelmingly disapproved of IPV (80.3%). Among the four indicators of empowerment, only the level of education differed according to parity. The proportion of women with a tertiary level of education was much higher among primiparous than among multiparous women (22.4% versus 19.2%). Conversely, the proportion of primiparous women with a primary level of education or less was less than half the proportion of multiparous women with a primary level of education (7.6% versus 16.7%).

**Table 1:** Indicators of empowerment among women^a^ by parity

		Primiparous (*n* = 578)	Multiparous (*n* = 765)	All (*n* = 1343)	
		%	%	%	*p*
Education level	Primary or less	7.6	16.7	12.8	**
	Secondary	58.9	64.1	61.8	
	Tertiary	33.4	19.2	25.3	
Worked in the previous 12 months	Worked for wages/salary	79.9	82.2	81.2	
	Did not work	20.1	17.8	18.8	
Own mobile phone	Has her own mobile phone	89.3	88.4	88.7	
	Does not have mobile phone	10.7	11.6	11.3	
Attitude towards intimate partner violence	Disapproves	80.1	80.4	80.3	
	Does not disapprove	19.9	19.6	19.7	

Data are from the 2014 Vietnam Multiple Indicator Cluster Survey (MICS).

^a^ women who had a singleton birth at a healthcare facility in the two years preceding the survey

** *p* ≤ 0.05, chi-square test

Among women who delivered in institutional settings, 14.8% underwent an ECS. This practice is more widespread among multiparous (18.4%) than among primiparous women (10.1%). ECS is linked to the level of empowerment only for multiparous women (Table 2). In this group, the use of ECS increases with all indicators of empowerment except for the recent working condition. Due to the absence of a relationship between work and empowerment for both groups, this indicator is not included in the following analyses.

**Table 2:** Rates of elective cesarean section in primiparous and multiparous women by maternal characteristics

		Primiparous (*n* = 578)		Multiparous (*n* = 765)		All (*n* = 1342)	
		%	*p*	%	*p*	%	*p*
Attitude towards intimate partner violence	Violence not justified	10.2		20.8	**	16.2	**
	Violence justified	9.6		8.0		9.1	
Education level	Primary or less	15.9		10.9	**	11.6	**
	Secondary	9.1		16.1		13.1	
	Tertiary	10.9		32.7		20.3	
Own mobile phone	Has her own mobile phone	10.1		20.0	**	15.7	**
	Does not have mobile phone	9.7		6.7		7.9	
Work in the previous 12 months	Worked for wages/salary	9.3		18.7		14.8	
	Did not work	12.9		16.9		15.1	
Number of antenatal care visits	3 or fewer	5.1	**	14.6	**	11.4	**
	4 to 7	8.7		15.5		12.5	
	8 or more	14.0		27.2		20.4	
Assistance at antenatal care	Doctor	9.9		18.3		14.6	
	No doctor	13.8		19.4		17.8	
Age at delivery (years)	15-19	5.2	**	14.3	*	5.8	**
	20-34	10.3		17.1		14.3	
	35-49	31.3		26.5		27.0	
Place of delivery	Public sector	9.9		17.9		14.4	*
	Private sector	15.8		25.7		23.6	
Perceived size of newborn	Average	8.1	**	18.3		13.8	*
	Larger than average	29.4		17.0		21.0	
	Smaller than average	8.5		21.0		15.6	
Sex of newborn	Female	8.8		18.4		14.2	
	Male	11.1		18.4		15.3	
Household	Poorest	6.9		10.4	**	8.3	**
Quintile of wealth	Poor	10.9		13.3		12.3	
	Middle	6.5		13.1		10.6	
	Rich	9.2		18.3		13.8	
	Richest	16.3		34.9		27.7	
Ethnicity of Household head	Kinh, Hoa	10.2		19.2	*	15.5	*
	Ethnic minority	9.3		10.8		10.0	
Place of Residence	Rural	8.2	**	12.5	**	10.7	**
	Urban	14.4		31.4		24.1	
Region	North-Central	12.7	*	16.6	**	15.1	**
	Red River Delta	6.3		18.3		13.8	
	North Midlands	13.8		12.9		13.5	
	Central Highlands	5.3		10.2		8.0	
	Southeast	13.9		27.8		21.6	
	Mekong River Delta	5.2		18.5		12.9	

Data are from the 2014 Vietnam Multiple Indicator Cluster Survey (MICS)

** *p *≤ 0.05, chi-square test

*: *p* ≤ 0.10, chi-square test

Among multiparous women, the rate of ECS was three times higher among those with a tertiary level of education than among those with a primary level of education or less (32.7% versus 10.9%). Similarly, this rate was three times higher among multiparous women who had their own mobile phone than among those who did not have their own mobile phone (20.0% versus 6.7%) and was more than twice as high among those who did not approve of IPV than among those who did not disapprove of it (20.8% versus 8.0%). In both groups, the use of ECS was linked to higher levels of childbirth medicalization. In particular, the use of ECS was much higher among women who had 8 ANC visits or more than among those who had 3 or fewer ANC visits. The other main factor common to both groups was the place of residence. Women who lived in urban areas had a much higher ECS rate than women who lived in rural areas.

Some factors were more specific to one group. Among primiparous women, the rate of ECS among those aged 35 years or older was threefold higher than the rate of ECS among women aged 20–34 years and sixfold higher than the rate of ECS among teenagers. The same multiplying factor applied to women who perceived their newborn’s size to be larger than average. Among multiparous women, those who lived in households that belonged to the richest quintile had a rate of ECS that was more than three times higher than the ECS rate of those who lived in households that belonged to the poorest quintile. The ECS rate is especially high in the Southeast region.

The main correlates of ECS were identified for each group separately. Among primiparous women (Table 3), after controlling for all factors except the factors that measure the influence of women’s empowerment (Model 1), the following three main factors were associated with the use of ECS: perceiving the size of the newborn as larger than average was strongly and positively associated with ECS (OR = 4.270, CI [2.249; 8.106]); being aged 35 years or older was also strongly associated with higher levels of ECS (OR = 4.654, CI [2.053; 10.553]); and living in the Red River Delta (OR = 0.344; CI [0.172; 0.689]) or the Mekong River Delta (OR = 0.282; CI [0.113; 0.705]) was strongly associated with a low level of ECS. When the indicators of empowerment were included in the analysis (Model 2), these trends remained. In addition, the likelihood of having ECS was halved for primiparous women who had 3 or fewer ANC visits.

**Table 3:** Multivariate analysis of the factors associated with elective cesarean section for primiparous women

	Model 1 (*n* = 578)		Model 2 (*n* = 578)	
	OR	95% CI	OR	95% CI
Attitude towards intimate partner violence (reference = violence justified)				
Violence not justified			0.962	0.499; 1.852
Education level (reference = secondary)				
Primary or less			2.318	0.647; 8.301
Tertiary			0.764	0.399; 1.462
Ownership of mobile phone (reference = has her own mobile phone)				
Does not have her own mobile phone			2.321	0.734; 7.337
Number of antenatal care visits (reference = 4 to 7)				
3 or fewer	0.543	0.261; 1.130	0.431**	0.189; 0.982
8 or more	1.517	0.850; 2.705	1.628	0.924; 2.869
Age at delivery (reference = 20-34 years)				
15-19	0.615	0.211; 1.791	0.523	0.191; 1.428
35-49	4.654**	2.053; 10.553	4.024**	1.538; 10.528
Place of delivery (reference = public sector)				
Private sector	2.265	0.647; 7.932	2.422	0.661; 8.876
Perceived size of newborn (reference = average)				
Larger than average	4.270**	2.249; 8.106	4.410**	2.241; 8.681
Smaller than average	1.159	0.468; 2.874	1.178	0.490; 2.835
Household quintile of wealth (reference = middle)				
Poorest	1.480	0.546; 4.012	0.957	0.332; 2.757
Poor	1.933	0.757; 4.937	1.970	0.723; 5.367
Rich	1.442	0.538; 3.864	1.629	0.572; 4.640
Richest	2.044	0.763; 5.472	2.780	0.903; 8.553
Ethnicity of household head (reference = Kinh, Hoa)				
Ethnic minority	1.029	0.504; 2.102	0.991	0.453; 2.170
Place of residence (reference = rural)				
Urban	1.089	0.554; 2.140	0.988	0.491; 1.990
Region (reference = North-Central)				
Red River Delta	0.344**	0.172; 0.689	0.361**	0.173; 0.755
North Midlands	1.224	0.592; 2.529	1.406	0.668; 2.960
Central Highlands	0.478	0.212; 1.078	0.486	0.198; 1.191
Southeast	0.638	0.264; 1.545	0.511	0.197; 1.331
Mekong River Delta	0.282**	0.113; 0.705	0.259**	0.103; 0.652

Model 1: Without agency variables; Model 2: With agency variables significant in the bivariate analysis

Data are from the 2014 Vietnam Multiple Indicator Cluster Survey (MICS).

** *p* ≤ 0.05

Among multiparous women (Table 4), place of residence and age at delivery were strongly associated with ECS. Living in urban areas doubled the likelihood of having ECS (OR = 2.103; CI [1.437; 3.079]). The link between the use of ECS and being aged 35 years or older was also strong (OR = 1.580; CI [1.005; 2.458]). One peculiar effect was the positive relationship between belonging to the richest household quintile and undergoing ECS. This association, which was relatively strong (OR = 2.355; CI [1.307; 4.244]) when the indicators of empowerment were not included (Model 1), was not significant when all factors were controlled by empowerment (Model 2). The only indicator of empowerment significantly linked to ECS was attitude towards IPV. Considering that IPV is not justified more than doubled the likelihood of using ECS (OR = 2.415; CI [1.234; 4.725]).

**Table 4:** Multivariate analysis of the factors associated with elective cesarean section for multiparous women

	Model 1 (*n* = 765)		Model 2 (*n *= 765)	
	OR	95% CI	OR	95% CI
Attitude towards intimate partner violence (reference = violence justified)				
Violence not justified			2.415**	1.234; 4.725
Education level (reference = secondary)				
Primary or less			0.750	0.405; 1.389
Tertiary			1.536	0.953; 2.476
Ownership of mobile phone (reference = has her own mobile phone)				
Does not have her own mobile phone			0.394	0.146; 1.063
Number of antenatal care visits (reference = 4 to 7)				
3 or fewer	1.268	0.782; 2.057	1.376	0.833; 2.273
8 or more	1.282	0.841; 1.955	1.293	0.836; 2.000
Age at delivery (reference = 20–34 years)				
15–19	1.230	0.584; 2.594	1.230	0.562; 2.696
35–49	1.580**	1.005; 2.485	1.673**	1.053; 2.658
Place of delivery (reference = public sector)				
Private sector	1.205	0.645; 2.251	0.113	0.584; 2.123
Perceived size of newborn (reference = average)				
Larger than average	0.907	0.566; 1.451	0.915	0.563; 1.487
Smaller than average	1.266	0.717; 2.238	1.322	0.760; 2.298
Household quintile of wealth (reference = middle)				
Poorest	0.924	0.403; 2.119	0.946	0.406; 2.202
Poor	1.131	0.611; 2.093	1.254	0.661; 2.380
Rich	1.301	0.657; 2.574	1.122	0.559; 2.251
Richest	2.355**	1.307; 4.244	1.633	0.851; 3.134
Ethnicity of household head (reference = Kinh, Hoa)				
Ethnic minority	0.812	0.447; 1.475	0.914	0.497; 1.681
Place of residence (reference = rural)				
Urban	2.103**	1.437; 3.079	2.004**	1.349; 2.977
Region (reference = North-Central)				
Red River Delta	0.984	0.566; 1.709	0.829	0.462; 1.488
North Midlands	0.911	0.498; 1.666	0.735	0.381; 1.417
Central Highlands	0.645	0.320; 1.303	0.674	0.335; 1.355
Southeast	1.226	0.703; 2.136	1.248	0.696; 2.237
Mekong River Delta	1.368	0.771; 2.429	1.290	0.707; 2.355

Model 1: Without agency variables; Model 2: With agency variables significant in the bivariate analysis

Data are from the 2014 Vietnam Multiple Indicator Cluster Survey (MICS)

** *p* ≤ 0.05

## Discussion

This paper examines the relationship between women’s empowerment and recourse to ECS as opposed to undergoing a trial of labor for a single pregnancy in Vietnam, a middle-income country with a rising level of biomedicalization in the last two decades [19]. By focusing on empowerment as an exposure variable, we identify the major trends regarding the influence of the level of education, employment, material resources, and attitudes towards IPV.

In 2014, the ECS rate in this country (14.8% of institutional births) represented slightly more than half of the overall CS rate (27.5%) [21]. This proportion was lower than the proportion observed at the population level in some neighboring middle-income countries. In Thailand, the proportion of women who have had ECS reached 21.2%, which represents two-thirds of the overall CS rate (32.7%) [29]. In Mongolia, the proportion of women who have had ECS (18.8%) is higher than that in Vietnam, whereas the overall CS rate (26.2%) is lower [30]. This specificity of the low proportion of ECS among all CS in Vietnam could be due to the implementation of social insurance that may encourage women to undergo CS and healthcare workers to underreport ECS. This outcome is linked to the rules of the social insurance regime, which limits coverage to medically indicated CS as stated in the successive laws on social protection during this period [31, 32]. This finding also suggests that despite high levels of ECS in Vietnam, there is still room for an additional increase in ECS.

In Vietnam, new norms of surveillance, increased knowledge, and extended health and biomedical responsibilities are being promoted in a stratified way [19]. State policies have encouraged the intensive use of technologies such as echography during ANC. This trend has been interpreted as revealing the difficulty of accepting uncertainty regarding the issue of pregnancy, a pitfall that exists in a nexus that links individual aspirations and state policies [33]. This interpretation is further updated and strengthened by a new emphasis on “population quality” [34]. In addition, specific sociocultural factors in favor of CS have been reported, such as a search for a propitious day [24] and a preference for sons in some areas of the country [35]. Women’s requests may result from a power dynamic between members of the women’s family and available economic resources at the household level. By making a distinction between the factors at the individual and household levels, our analysis helps to disentangle part of these influences, as shown by the impact of the household level of wealth and women’s attitudes towards IPV.

IPV has become a topic of national concern in Vietnam [36]. However, certain policies tend to increase gender-related violence towards women [37] despite the adoption of recent laws in favor of gender equity and against violence towards women [38]. As a consequence, underlying gender and power imbalances lead to IPV [39], and women have little “possibility to make independent decisions about their own reproductive health” in rural areas [40]. Overall, a majority of ever-partnered women (58%) have experienced at least one type of physical, sexual or emotional violence, and a high proportion of them (27%) have experienced domestic violence in the last 12 months [36]. More qualitative investigations show that emotional violence, which is the most common type of violence, is experienced by a large proportion of pregnant women [41]. Emotional violence leads to psychological distress and suffering and may cause adverse health outcomes [41]. The proportion of women who approve of IPV is higher in the oldest generations [39]. This may be due to the pervasiveness of unequal gender norms, a perception of women’s dependency on the husband, especially when the couple has children, and a tendency to report community norms rather than to express their own personal attitudes [39]. However, new trends in favor of more equal gender norms are developing, partly due to program prevention [39]. In this context, the refusal of IPV as measured in our study is an indicator of women’s ability to be more self-confident and assertive, to discuss and defend their choice of undergoing ECS and to take action.

Another factor of women’s empowerment is the use of mobile phones. This occurs in a context where the use of digital equipment has increased rapidly and has become widespread [42]. This trend influences health-related behavior, as shown in a study that demonstrated the influence of online comments on the perceptions of health risk issues [43].

The absence of the effect of women’s work on ECS may be due to several factors. First, given that we focused on women who delivered in the last 2 years, some women may have stopped their economic activity because of pregnancy complications. Therefore, economic activity may not strictly reflect their level of empowerment. Second, a large proportion of women benefit from the positive effect of health coverage, which alleviates financial constraints [44]. In 2012, 55% of women of reproductive age had health insurance [19]. Third, working conditions are heterogeneous. Further investigation that contrasts the influence of the type and sector of activity could help us better understand the relationship between economic activity and the mode of delivery.

The impact of education level disappeared when the effect of education was controlled by other correlates.

The ECS rate was higher among multiparous (18.4%) than among primiparous women (10.1%). This trend is opposite to that observed for the overall rate of CS in both rural areas (42.4% among multiparous vs. 45.5% among primiparous) and urban areas (20.5% among multiparous vs. 25.2% among primiparous) [45]. This finding can be partly explained by repeated CS. Indeed, despite the possibility of performing successful vaginal delivery after CS in Vietnam [46], almost all women who have previously had CS undergo another CS [19]. Further exploration of our database shows that most multiparous women had only one child (76.2%), approximately two-thirds had a previous birth interval of 4 years or more (63%), and the estimated mean length of the interval between the last two births was 4 years. Given that the CS rate reached 20.0% in 2011 [47], we cannot rule out the possibility that repeated CS plays a major role in the difference in the ECS rate between primiparous and multiparous women. This cumulative effect from one childbirth to the next underlines the need to focus efforts on reducing non-medically indicated CS in primiparous women.

One striking feature was the difference in the relationship between women’s empowerment and the use of ECS according to parity. Among primiparous women, no indicator of empowerment was associated with higher rates of ECS, whereas among multiparous women, all indicators were positively associated with higher rates of ECS except work in the previous 12 months.

Considering women’s attitudes towards IPV helped us not only measure some of the effects of women’s empowerment on ECS but also better specify the impacts of the other correlates. Two correlates deserve greater attention, namely, the household level of wealth in multiparous women and the number of ANC visits in primiparous women. A positive relationship between living in the richest quintile of households and a higher rate of CS has been observed in many areas, including Vietnam [48]. Our findings reveal that a similar trend applies to ECS, but this positive impact is mitigated by the effect of women’s intrinsic agency as measured by their attitudes towards IPV. This is especially true of multiparous women. In this group, once attitudes towards IPV were considered, there was no longer a significant difference between women who live in the richest household quintile and women who live in households of the middle-range wealth quintile. The contrasting effect of women’s perception of IPV on childbirth according to parity can be explained by the fact that women’s feeling of dependency towards their husbands is fostered by having had children with him, as shown in a qualitative study on women’s perceptions of IPV in Vietnam [49].

Among primiparous women, the negative effect of having had 3 ANC visits or less appeared only when the indicators of women’s empowerment were taken into account. This result is due to two phenomena. First, the likelihood of using ECS increased with a higher number of ANC visits and a higher level of women’s empowerment. Second, the proportion of women who disapproved of IPV violence was higher among those who had 8 ANC visits or more (86.0%) than among women who had 4 to 7 visits (79.2%) or 3 antenatal visits or less (69.4%). As a result, controlling the effect of ANC visits with women’s agency allows us to better distinguish between the medical and social factors of ECS.

To be more specific about the potential link between the effect of the household level of wealth and especially its potential influence in terms of women’s empowerment, we tested another model (Model 2) that excluded the household level of wealth. The results show that the main trends remain in both parity groups. In particular, the effect of attitude towards IPV remains at the same level in multiparous women (OR = 2.383; CI [1.225; 4.636] in the model without the variable vs. OR = 2.415; CI [1.234; 4.725] when the household level of wealth is considered), and it remains nonsignificant in primiparous women. The only variables affected by the exclusion of the household level of wealth are the level of education in multiparous women and the number of ANV visits in primiparous women. In multiparous women, when the household level of wealth was not taken into account, having reached a tertiary level of education became significant and associated with a higher likelihood of undergoing ECS (OR = 1.778; CI [1.168; 2.706]). In primiparous women, having attended fewer than 3 ANC visits became nonsignificant. These results show that considering the attitude towards IPV allows us to identify a significant factor of the recourse to ECS.

Although not analyzed as such, some correlates involved some degree of women’s empowerment. This was the case for age, which was the only correlate for which the effect remained significant in both groups even after considering women’s empowerment. In a society where an older age places an individual in a position of respect and authority in everyday relationships [50], we can expect women over 35 years to be more prone to discuss their choice with healthcare workers, their partner and their relatives. Our results show that women aged 35 years and over were twice as likely as women aged 20–34 years to have recourse to ECS. However, this trend could also involve a lack of empowerment. Women with a later age at delivery tend to be stigmatized due to the high value attributed to fertility, marriage and Confucian values marked by women’s submission to the patriarchal order [22]. Furthermore, national clinical guidelines explicitly mention age 35 years or older as an age when pregnant women need more regular follow-up and more intensive prenatal screening than “normal pregnancies” [27]. This exemplifies the multiple and sometimes contradictory effects of empowerment. The part of empowerment that these effects relate to remains to be explored. Age could be mainly be linked to instrumental agency, in particular, regarding women’s ability to make household and family-level decisions. To document this aspect, more specific questions must be collected and analyzed.

Among primiparous women, the perception of the size of their newborn as above average increases the likelihood of using ECS. This may be favored by a link between the size and weight of the newborn, given that Vietnam’s national guidelines use an upper limit of 3.5 kg for the average weight of the fetus instead of 4.0 or 4.5 kg, as in many other countries and international norms [27, 51, 52].

Given the almost systematic recourse to ECS for the women who previously had CS as mentioned above, the positive impact of living in urban areas on the higher level of ECS in multiparous women may be explained only by the catch-up phenomenon between rural and urban areas that leads to a convergence of the CS rates. Indeed, many multiparous women who had an ECS were primiparous women who had a CS at a time when the gap between rural and urban areas was larger than it currently is [45]. However, more data are needed to determine whether this trend in the CS rates applies to the ECS rates.

For primiparous women, the region has a significant effect, whereas this is not true for multiparous women. Two regions in particular were marked by low levels of ECS, specifically, the Red River Delta in the northeastern part of the country and the Mekong River Delta in the southwest. A more in-depth investigation of the impact of other factors linked to local beliefs regarding the mode of delivery and the level of healthcare equipment could allow us to better understand these differences.

The relationship between ECS and empowerment can also be bidirectional. Women’s empowerment may foster the use of ECS, and, conversely, having experienced delivery may increase women’s empowerment, as shown in other areas [53]. Despite the possible negative effects on intrinsic agency as stated before, such a causal link is consistent with the analyses of gender in Vietnamese society [22].

This study has limitations. First, ECS may be decided by healthcare workers without asking the woman’s opinion. ECS may also be because of a woman’s request. Our data do not provide information on this issue. However, both circumstances involve women’s empowerment. Second, among the multiparous women, we cannot identify those who previously had CS. This would help us to better select women according to how their empowerment would influence their decision. Third, limitations to drawing conclusions exist in the cross-sectional nature of this study, which implies that the direction of associations cannot be established. However, the women expressed their current attitudes regarding IPV and referred to the most recent birth in the previous two years, which limited the biases. Fourth, our empowerment variables do not reflect the entire potential effect of this complex phenomenon. Despite these limitations, we believe that this study emphasizes important trends to better understand the overall phenomenon of rising ECS rates and, more broadly, some actual and potential forms and effects of women’s empowerment.

## Conclusions

This study provides new insights into issues regarding women’s empowerment and the biomedicalization of childbirth. Our results underline the need for a multidisciplinary approach that mobilizes social science and the biomedical sciences to better document the impact of women’s empowerment on the mode of delivery.

Our results suggest that both the ECS rate and the share of ECS among all CS should be closely monitored and that parity is a major characteristic to consider in programs that aim to control ECS. In particular, among multiparous women, an equitable view of gender roles is correlated with undergoing ECS rather than undergoing a trial of labor. This is particularly pertinent in the context of Goals 3 and 5 of the 2030 Agenda for Sustainable Development that call for improved health, well-being and gender equality.

Based on these trends, specific modes of nonclinical interventions from healthcare workers could be developed during ANC that aim to deliver more accurate information to pregnant women. In particular, providing more in-depth information about the advantages and drawbacks of CS versus vaginal delivery could help to reduce the fear of vaginal delivery and consequently contribute to better controlled ECS rates.

## Data Availability

The datasets analyzed during the current study are freely available through the UNICEF MICS website [https://mics.unicef.org/surveys].

## Abbreviations

ANC: Antenatal care
CS: Cesarean section
ECS: Elective cesarean section
IPV: Intimate partner violence
OR: Odds ratio
